# The Story of the Insane from Year to Year

**Published:** 1897-12-18

**Authors:** 


### THE STORY OF THE INSANE FROM YEAR TO YEAR.

Tenth Series.

### INVERNESS DISTRICT ASYLUM.

An increase from 468 to 518 has to be chronicled during the year. There were 121 first admissions, 45 readmissions, 83 recoveries, and 35 deaths. The recoveries stand at 50 per cent. on the admissions. This is certainly a high percentage, but then the readmissions are also high. The deaths 7·1 per cent. on the average number resident. Only four of the deaths were caused by cerebral diseases, but there were 10 deaths from phthisis, 4 from pneumonia, and 5 from pleurisy. Of the year's admissions 41 owed their insanity to various moral causes, 24 to intemperance in drink, and 63 to hereditary influence. Dr. Keay draws attention to the fact that patients are kept at home for months or years owing to the expense of sending them to the asylum. Dr. Keay says this is not economy, and everyone engaged in treating insane patients knows that the sooner they come under care the greater will be the chances of recovery. "The patient is sent to the asylum when hopelessly insane, and remains for the rest of his life a burden on the parish." This is true enough, but then the burden on the parish dies with him. Had he been sent to the asylum immediately on becoming insane he would have been discharged, probably, in a few months; the insanity would have recurred, and he would have been admitted and discharged again and again, and after each "recovery" he would probably have had insane children, so that in the next generation the parish would have been saddled with two or three additional lunatics. As a matter of "economy" we do not think Dr. Keay is right. His own report shows that more than one-third of the admissions were hereditary, and even this high proportion is most likely below the truth, if the truth could be arrived at. The asylum is more than full, but additions are being made which will relieve the overcrowding. It is to this overcrowding that Dr. Keay attributes the high proportion of pulmonary diseases in the causes of death. The nurses are being trained by the medical officers, and thirteen have obtained the nursing certificates of the Medico-Psychological Association. Dr. Keay gives the names of those who passed. He does not tell us how many failed; but he says "that the examination is a really searching test of nursing knowledge." In view of the correspondence which lately appeared in the medical papers we think this is questionable; but that a step in the right direction has been taken is unquestionable, and we hope the staff of the Inverness Asylum will follow the road onwards.

